# Draft Genome Sequence of *Streptomyces* sp. Strain ventii, Isolated from a Microbial Mat near Hydrothermal Vents within the Axial Seamount in the Pacific Ocean, and Resequencing of the Type Strains Streptomyces lonarensis NCL 716 and Streptomyces bohaiensis 11A07

**DOI:** 10.1128/MRA.00607-20

**Published:** 2020-08-06

**Authors:** Rachel M. Loughran, Edward A. Mitchell, Oliver B. Vining, David A. Gallegos, Monica C. Deadmond, Benjamin J. Wasson, Kaysa M. Pfannmuller, Brie E. Paddock, Marc J. Koyack, David K. Oline, Blake Ushijima, Jimmy H. Saw, Kerry L. McPhail, Patrick Videau

**Affiliations:** a Department of Biology, Southern Oregon University, Ashland, Oregon, USA; b Department of Pharmaceutical Sciences, Oregon State University, Corvallis, Oregon, USA; c Department of Chemistry, Southern Oregon University, Ashland, Oregon, USA; d Smithsonian Marine Station, Fort Pierce, Florida, USA; e Department of Biological Sciences, The George Washington University, Washington, DC, USA; Broad Institute

## Abstract

The draft genome of *Streptomyces* sp. strain ventii, an environmental isolate recovered from deep-sea hydrothermal vents in the Pacific Ocean, is presented along with the resequenced draft genomes of the type strains Streptomyces bohaiensis 11A07 and Streptomyces lonarensis NCL 716.

## ANNOUNCEMENT

Members of the genus *Streptomyces* are Gram-positive, spore-forming, filamentous bacteria that often synthesize desirable antimicrobials, cytotoxins, and other lead compounds (1–4). The type strains Streptomyces bohaiensis 11A07 and Streptomyces lonarensis NCL 716 produce antimicrobials and an α-amylase, respectively (5–7). *Streptomyces* sp. strain ventii was isolated from the Juan de la Fuca Ridge in the Northeast Pacific Ocean. The draft genome of *Streptomyces* sp. ventii is presented, along with the resequenced draft genomes of *S. bohaiensis* 11A07 and *S. lonarensis* NCL 716.

Deep-sea samples were collected during the 2011 New Millennium Observatory expedition, run through the National Oceanic and Atmospheric Administration (NOAA) Vents Program at Oregon State University and the NOAA Pacific Marine Environmental Laboratory. A microbial mat near hydrothermal vents on the Axial Seamount (46.06°N, 130°W) at a depth of 2,190 m was collected with a custom syringe-based sampler on the remotely operated vehicle (ROV) Jason II (aboard the research vessel [R/V] *Thompson*). The sample was diluted 1:1,000 in sterile Instant Ocean, spread onto 1/10 Zobell marine agar 2216 with sterile swabs, and incubated at 28°C for 2 weeks. Strain maintenance was performed on International Streptomyces Project 2 (ISP2) medium supplemented with 0.1 M sodium phosphate buffer to a pH of 8.0 (buffered ISP2) at 28°C (8). Strains 11A07 (DSM 42125) and NCL 716 (DSM 42084) were obtained from the Leibniz Institute DSMZ and cultured on buffered ISP2 medium at 28°C. *Streptomyces* sp. ventii was confirmed as a member of the *Streptomyces* genus through 16S rRNA gene sequencing and BLAST analysis (9, 10). Following a 4-day incubation at 28°C in buffered ISP2 broth shaken at 120 rpm, DNA was isolated by phenol-chloroform extraction (11). The raw reads were obtained from the Microbial Genome Sequencing Center, LLC (Pittsburgh, PA), using 151-bp paired-end read libraries prepared with the Illumina Nextera kit (12). Libraries were run on the Illumina NextSeq 550 platform yielding 9,643,560, 12,101,213, and 14,343,200 pairs of raw reads for strains ventii, 11A07, and NCL 716, respectively. FastQC was used to assess the read quality (http://www.bioinformatics.babraham.ac.uk/projects/fastqc/); adapter sequence removal, read quality trimming (removal and trimming parameters: ktrim = r, ordered, minlen = 50, mink = 11, tbo, rcomp = f, k = 21, ow = t, ftm = 5, zl = 4, qtrim = rl, trimq = 20), and analysis were performed using BBDuk in the BBMap package (http://sourceforge.net/projects/bbmap/), and genomes were assembled with SPAdes v. 3.14.0 using the “–careful” option and specifying kmers of 21, 33, 55, 77, 99, and 121 (13). Contigs and scaffolds greater than 1,000 bp were retained for analysis. Assemblies were analyzed with the Prokaryotic Genome Annotation Pipeline (PGAP), and DNA-DNA hybridization (DDH) was performed *in silico* using the DSMZ Genome-to-Genome Distance Calculator with default settings (14, 15).

The relevant genome characteristics are presented in Table 1. The resequenced *S. bohaiensis* and *S. lonarensis* genomes presented here are derived from the same strains (DDH = 99.50% and 99.40%, respectively, between the two versions of each genome) and display higher mean coverage in fewer contigs with more of the genomes in scaffolds greater than 50 kb (Table 1). Removal of contigs and scaffolds smaller than 1,000 bp from the published assemblies did not greatly alter their quality or statistics (16).

**TABLE 1:** Characteristics of the draft genome sequences from the *Streptomyces* strains described in this work and the previously published Streptomyces bohaiensis strain 11A07 and Streptomyces lonarensis strain NCL 716 genome sequences^*a*^

Relevant characteristic	Data for strain:						
	*Streptomyces* sp. ventii	*Streptomyces bohaiensis* 11A07	*Streptomyces lonarensis* NCL 716	*Streptomyces bohaiensis* 11A07^*b*^	*Streptomyces bohaiensis* 11A07 (>1,000 bp)^*b*^,^*c*^	*Streptomyces lonarensis* NCL 716^*b*^	*Streptomyces lonarensis* NCL 716 (>1,000 bp)^*b*^,^*c*^
Genome size (bp)	5,708,881	5,631,365	5,953,444	5,698,492	5,621,459	6,006,371	5,884,873
G+C content (%)	73.34	73.75	73.79	73.82	73.81	73.83	73.81
No. of scaffolds	474	547	482	883	649	1,154	745
No. of contigs	486	565	502	890	656	1,162	753
*N*_50_ (bp)	19,934	16,815	20,580	13,470	13,894	11,176	11,454
Mean coverage (fold)	67.9	72.6	86.7	13	13	14	14
No. of genes annotated with PGAP (15)	4,842	4,919	5,133	NA	NA	NA	NA
No. of RNAs from PGAP annotation	66	72	72	NA	NA	NA	NA
% of genome in scaffolds >50 kb	14.95	10.25	13.50	0.91	0.92	0.87	0.89
GenBank accession no. (version no.)	JAAVJB000000000 (JAAVJB010000000)	JAAVJC000000000 (JAAVJC010000000)	JAAVJD000000000 (JAAVJD010000000)	BHZH00000000 (BHZH00000000.1)	NA	BHZG00000000 (BHZG00000000.1)	NA
BioSample accession no.	SAMN14445373	SAMN14448217	SAMN14448297	SAMD00146571	NA	SAMD00146572	NA
SRA accession no.	SRS6438928	SRS6447757	SRS6447181	NA	NA	NA	NA

a The published assemblies were filtered with BBMap to remove scaffolds and contigs smaller than 1,000 bp, as was done for genome sequences presented in this work, and analyzed with the same software.

b Data taken from Terahara et al. (16).

c Data for the genome assemblies with all contigs/scaffolds <1,000 bp removed.

### Data availability.

The whole-genome shotgun projects, BioSample material, and raw reads have been deposited in DDBJ/ENA/GenBank under the accession numbers listed in Table 1.

## References

[1] Chater KF 2016 Recent advances in understanding *Streptomyces*. F1000Res 5:2795. doi:10.12688/f1000research.9534.1. 27990276 PMC5133688

[2] Dharmaraj S 2010 Marine *Streptomyces* as a novel source of bioactive substances. World J Microbiol Biotechnol 26:2123–2139. doi:10.1007/s11274-010-0415-6.

[3] Procópio REDL, Da Silva IR, Martins MK, De Azevedo JL, De Araújo JM 2012 Antibiotics produced by *Streptomyces*. Braz J Infect Dis 16:466–471. doi:10.1016/j.bjid.2012.08.014. 22975171

[4] Sivalingam P, Hong K, Pote J, Prabakar K 2019 Extreme environment *Streptomyces*: potential sources for new antibacterial and anticancer drug leads? Int J Microbiol 2019:5283948. doi:10.1155/2019/5283948. 31354829 PMC6636559

[5] Pan H-Q, Cheng J, Zhang D-F, Yu S-Y, Khieu T-N, Son CK, Jiang Z, Hu J-C, Li W-J 2015 *Streptomyces bohaiensis* sp. nov., a novel actinomycete isolated from *Scomberomorus niphonius* in the Bohai Sea. J Antibiot (Tokyo) 68:246–252. doi:10.1038/ja.2014.137. 25269462

[6] Sharma TK, Mawlankar R, Sonalkar VV, Shinde VK, Zhan J, Li W-J, Rele MV, Dastager SG, Kumar LS 2016 *Streptomyces lonarensis* sp. nov., isolated from Lonar Lake, a meteorite salt water lake in India. Antonie Van Leeuwenhoek 109:225–235. doi:10.1007/s10482-015-0626-9. 26597560

[7] Sharma TK, Bhadane VA, Kumar LS, Rele MV, Bhawar G, Rahman I 2013 Optimization of the production of a maltooligosaccharides producing amylase from the alkalophilic *Streptomyces lonarensis* strain NCL 716 using SVR modeling. Starke 65:179–185. doi:10.1002/star.201200094.

[8] Shirling EB, Gottlieb D 1966 Methods for characterization of *Streptomyces* species. Int J Syst Evol Microbiol 16:313–340. doi:10.1099/00207713-16-3-313.

[9] Altschul SF, Gish W, Miller W, Myers EW, Lipman DJ 1990 Basic local alignment search tool. J Mol Biol 215:403–410. doi:10.1016/S0022-2836(05)80360-2. 2231712

[10] Lane DJ 1991 16S/23S rRNA sequencing, p 115–175. *In* Stackebrandt E, Goodfellow M (ed), Nucleic acid techniques in bacterial systematics. Wiley, Chichester, NY.

[11] Sambrook J, Russell DW 2001 Molecular cloning: a laboratory manual. Cold Spring Harbor Laboratory, Cold Spring Harbor, NY.

[12] Baym M, Kryazhimskiy S, Lieberman TD, Chung H, Desai MM, Kishony R 2015 Inexpensive multiplexed library preparation for megabase-sized genomes. PLoS One 10:e0128036. doi:10.1371/journal.pone.0128036. 26000737 PMC4441430

[13] Loughran RM, Esquivel AR, Deadmond MC, Koyack MJ, Paddock BE, O’Hanlon SM, Ushijima B, Saw JH, Videau P 2020 Draft genome sequence of *Vibrio* sp. strain OCN044, isolated from Palmyra Atoll, Northern Line Islands. Microbiol Resour Announc 9:e00042-20. doi:10.1128/MRA.00042-20. 32193232 PMC7082451

[14] Meier-Kolthoff JP, Auch AF, Klenk H-P, Göker M 2013 Genome sequence-based species delimitation with confidence intervals and improved distance functions. BMC Bioinformatics 14:60. doi:10.1186/1471-2105-14-60. 23432962 PMC3665452

[15] Tatusova T, DiCuccio M, Badretdin A, Chetvernin V, Nawrocki EP, Zaslavsky L, Lomsadze A, Pruitt KD, Borodovsky M, Ostell J 2016 NCBI Prokaryotic Genome Annotation Pipeline. Nucleic Acids Res 44:6614–6624. doi:10.1093/nar/gkw569. 27342282 PMC5001611

[16] Terahara T, Naemura T, Nampo Y, Kobayashi T, Imada C, Hamada M, Tamura T 2019 *Streptomyces otsuchiensis* sp. nov., a biosurfactant-producing actinobacterium isolated from marine sediment. Int J Syst Evol Microbiol 69:3740–3744. doi:10.1099/ijsem.0.003638. 31693473

